# Correction: Maning et al. Antagonistic Roles of GRK2 and GRK5 in Cardiac Aldosterone Signaling Reveal GRK5-Mediated Cardioprotection via Mineralocorticoid Receptor Inhibition. *Int. J. Mol. Sci.* 2020, *21*, 2868

**DOI:** 10.3390/ijms22157767

**Published:** 2021-07-21

**Authors:** Jennifer Maning, Katie A. McCrink, Celina M. Pollard, Victoria L. Desimine, Jennifer Ghandour, Arianna Perez, Natalie Cora, Krysten E. Ferraino, Barbara M. Parker, Ava R. Brill, Beatrix Aukszi, Anastasios Lymperopoulos

**Affiliations:** 1 Laboratory for the Study of Neurohormonal Control of the Circulation, Department of Pharmaceutical Sciences, College of Pharmacy, Nova Southeastern University, Fort Lauderdale, FL 33328, USA; jm3706@mynsu.nova.edu (J.M.); km1911@mynsu.nova.edu (K.A.M.); cp1743@mynsu.nova.edu (C.M.P.); vd359@mynsu.nova.edu (V.L.D.); jg2901@mynsu.nova.edu (J.G.); ap2491@mynsu.nova.edu (A.P.); nc1174@mynsu.nova.edu (N.C.); kf713@mynsu.nova.edu (K.E.F.); barbaramparker@gmail.com (B.M.P.); avabrill@gmail.com (A.R.B.); 2 Department of Chemistry and Physics, Halmos College of Natural Sciences and Oceanography, Nova Southeastern University, Fort Lauderdale, FL 33328, USA; ba285@nova.edu

The authors wish to make the following correction to this paper [1]:

In the original article, there was a mistake in Figure 4A, as published. During the representative image assembly, a few images were inadvertently duplicated. This error occurred because of different magnification and exposure settings used among the five separate, independent experiments performed (in total) for this experimental series (TUNEL experiments), which caused this erroneous duplication to be overlooked. The corrected Figure 4 appears below. The authors apologize for any inconvenience caused and state that none of the results or conclusions of the paper are affected. The original article has been updated accordingly.

**Figure 4:**
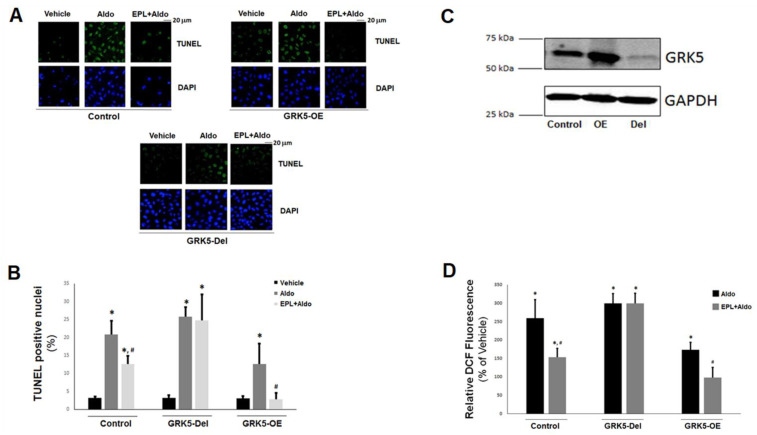
Effects of GRK5 in Aldo-induced apoptosis in adult rat ventricular myocytes (ARVMs): (**A**,**B**) Apoptotic cell death, measured by terminal deoxynucleotidyl transferase dUTP nick-end labeling (TUNEL), in cultured ARVMs having GRK5 genetically (via CRISPR) deleted (GRK5-Del) or overexpressing GRK5 (GRK5-OE) and treated with 100 nM Aldo or 100 nM Aldo in the presence of 10 µM eplerenone (EPL + Aldo) for 24 h. Representative images of TUNEL-positive nuclei identified by 4′,6-diamidino-2-phenylindole (DAPI) counterstaining are shown in (**A**), and the quantitation of the TUNEL imaging results are shown in (**B**). Control: CRISPR-negative control virus (CNCV)-infected cells. * *p* < 0.05, vs. vehicle; ^#^, *p* < 0.05, vs. Aldo; *n* = 5 independent experiments per transfection/treatment. (**C**) Immunoblotting for GRK5 in extracts from cultured ARVMs, transfected with control empty vector/mock lentivirus (Control), full-length wild-type GRK5-encoding lentivirus to overexpress GRK5 (OE), or CRISPR rat GRK5-specific lentivirus to delete the gene for GRK5 (Del). Total protein extracts were prepared 48 h post-infection and then separated on a 4–20% SDS-PAGE gel. A representative blot is shown, including GAPDH as loading control, of five independent experiments performed in duplicate, confirming GRK5 overexpression and deletion in OE and Del ARVMs, respectively. (**D**) Reactive oxygen species (ROS) generation, as measured with a 2′,7′-dichlorofluorescein diacetate (DCFDA)-based assay kit, in cultured ARVMs having GRK5 genetically (CRISPR-mediated) deleted (GRK5-Del) or overexpressing GRK5 (GRK5-OE) and treated with 100 nM aldosterone (Aldo) or 100 nM aldosterone in the presence of 10 µM eplerenone (EPL + Aldo) for 24 h. Results are expressed as % of the fluorescence measured upon vehicle (DMSO) treatment for each cell clone. Control: Empty vector/mock lentivirus-infected ARVMs. Eplerenone alone had no effect. * *p* < 0.05, vs. vehicle; ^#^ *p* < 0.05, vs. Aldo; *n* = 6 independent experiments per transfection/treatment.
